# Changes in hemodynamics, renal blood flow and urine output during continuous renal replacement therapies

**DOI:** 10.1038/s41598-020-77435-x

**Published:** 2020-11-27

**Authors:** S. N. Fernández, M. J. Santiago, R. González, J. López, M. J. Solana, J. Urbano, J. López-Herce

**Affiliations:** 1 Pediatric Intensive Care Department, Gregorio Marañón General University Hospital, Madrid, Spain; 2 Department of Pediatrics. School of Medicine, Complutense University of Madrid, Madrid, Spain; 3 Health Research Institute of the Gregorio Marañón Hospital, Madrid, Spain

**Keywords:** Acute kidney injury, Continuous renal replacement therapy

## Abstract

Continuous renal replacement therapies (CRRT) affect hemodynamics and urine output. Some theories suggest a reduced renal blood flow as the cause of the decreased urine output, but the exact mechanisms remain unclear. A prospective experimental study was carried out in 32 piglets (2–3 months old) in order to compare the impact of CRRT on hemodynamics, renal perfusion, urine output and renal function in healthy animals and in those with non-oliguric acute kidney injury (AKI). CRRT was started according to our clinical protocol, with an initial blood flow of 20 ml/min, with 10 ml/min increases every minute until a goal flow of 5 ml/kg/min. Heart rate, blood pressure, central venous pressure, cardiac output, renal blood flow and urine output were registered at baseline and during the first 6 h of CRRT. Blood and urine samples were drawn at baseline and after 2 and 6 h of therapy. Blood pressure, cardiac index and urine output significantly decreased after starting CRRT in all piglets. Renal blood flow, however, steadily increased throughout the study. Cisplatin piglets had lower cardiac index, higher vascular resistance, lower renal blood flow and lower urine output than control piglets. Plasma levels of ADH and urine levels of aquaporin-2 were lower, whereas kidney injury biomarkers were higher in the cisplatin group of piglets. According to our findings, a reduced renal blood flow doesn’t seem to be the cause of the decrease in urine output after starting CRRT.

## Introduction

Acute kidney injury (AKI) is a major complication in critically ill children in terms of incidence and outcomes, as it can affect about one out of three children that are admitted to the PICU. Reported prevalence of AKI requiring renal replacement therapies (RRT) ranges between 3 and 13.5% and a mortality rate of 30–50%. Up to 15% of survivors remain dialysis dependent^1–6^. The goal of RRT is to assist renal function by maintaining water and electrolyte balance. There are, basically, three modalities of RRT in pediatric patients: peritoneal dialysis, intermittent hemodialysis and continuous renal replacement therapies. Clinical evidence on which modality is best in terms of survival or renal recovery is not strong enough to make a definite statement on behalf of one or the other. Nevertheless, the latest evidence seems to be tipping the scale in favor of continuous veno-venous renal replacement therapies (CRRT) such as hemofiltration (CVVHF) or hemodiafiltration (CVVDHF), as they are better tolerated in hemodynamically unstable patients and allow gradual, continuous and programmed fluid removal^7–10^. For this reason, the use of CRRT in pediatric critical care units has increased immensely in the last two decades^11,12^.

CRRT-related hypotension or CRRT-related hemodynamic instability (CRRT-HI) is a relatively common complication^10,13–15^. Our research group has been studying the possible mechanisms leading to hypotension during the connection to CRRT for several years. A previous study revealed that initiating CRRT was accompanied by a significant decrease in arterial blood pressure, cardiac index and urine output (UO) in hemodynamically stable piglets^15^. Normal physiological responses to hypotension such as tachycardia and increased peripheral vascular resistance seemed to be impaired during connection. Renal blood flow, however, steadily increased throughout the observation period. This decrease in (UO) after starting CRRT is also observed in the clinical setting in pediatric patients, but has never been addressed directly in the scientific literature.

In the present study, we compared the impact of CRRT on hemodynamics, renal perfusion, UO and renal function in animals with normal renal function and in those with non-oliguric acute kidney injury (AKI).

## Results

Thirty-two healthy 2-to-3-month-old minipigs were studied. Mean weight was 9.1 kg (SD 1.6) and 54% were males. Hemodynamic and renal blood flow parameters were normal at baseline, and there were no statistical differences between groups, except in UO, which was higher in control piglets (Table 1).

**Table 1:** Baseline parameters.

	Control	Cisplatin	p
	Mean (SD)	Mean (SD)	
Temperature (°C)	38.7 (1.5)	37.2 (2.0)	0.22
Heart rate (bpm)	116 (29)	107 (25)	0.5
Systolic arterial pressure (mmHg)	115 (16)	112 (21)	0.35
Diastolic arterial pressure (mmHg)	65 (13)	63 (20)	0.15
Mean arterial pressure (mmHg)	86 (13)	82 (21)	0.21
Central venous pressure (cmH_2_O)	9 (3.7)	9 (4.3)	0.37
Cardiac index (L/min/m^2^)	4.09 (1.73)	3.34 (1.02)	0.42
Systemic vascular resistance index (dyn.sec/cm^5^/m^2^)	1605 (490)	1856 (527)	0.97
Renal blood flow (ml/min)	33.1 (12.3)	29.5 (16.9)	0.5
Resistive index (RI)	0.67 (0.11)	0.68 (0.1)	0.96
Pulsatility index	1.25 (0.37)	1.2 (0.37)	0.73
Urine output (ml/h)	45 (20)	25 (18)	0.05

*SD *Standard deviation, *Bpm *beats per minute.

### Renal perfusion parameters

**Figure 1:**
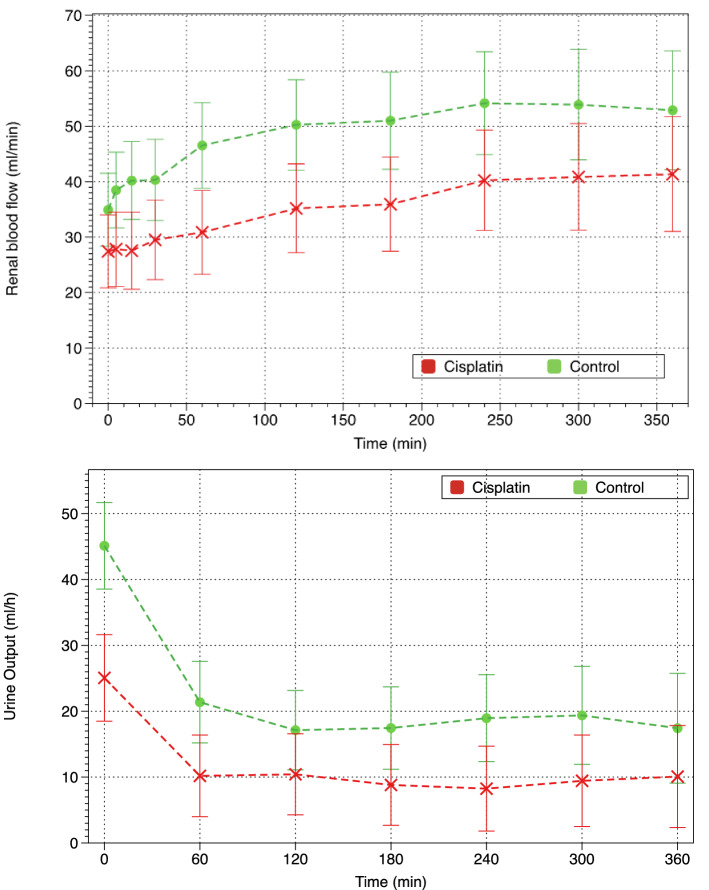
Evolution of renal blood flow and urine output.

Renal blood flow steadily increased throughout the study in all piglets (p < 0.001), but it was significantly lower in the cisplatin group (p = 0.015). Conversely, UO decreased immediately after starting CVVHDF, but it was significantly lower in cisplatin piglets (p = 0.002) (Fig. 1).

### Renal Doppler resistive index (RRI) and pulsatility index (PI)

Doppler RRI was normal at baseline in both groups (0.69 and 0.68, respectively. p = 0.88), and there were no significant differences in RRI after starting CVVHDF in any of the groups (p = 0.62).

### Hemodynamic parameters

**Figure 2:**
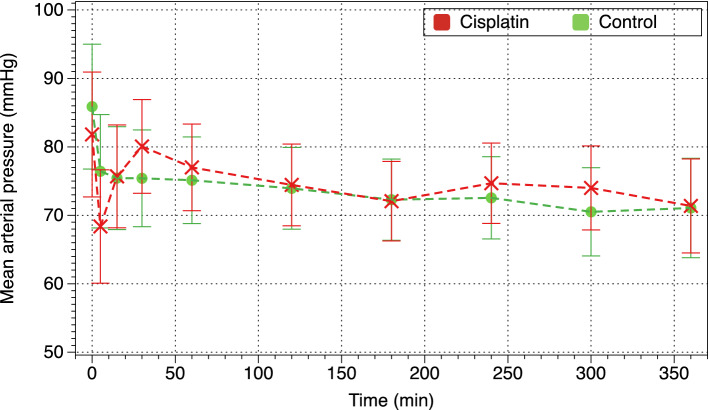
Evolution of mean arterial pressure.

Blood pressure decreased significantly during the first 5 min of therapy in both groups, and remained low throughout the study (Fig. 2). There were no differences between cisplatin and control piglets. Heart rate and temperature decreased in a similar fashion in both groups and, even though these differences did not reach statistical significance, temperature was much lower (almost 1.5 °C) in cisplatin piglets.

**Figure 3:**
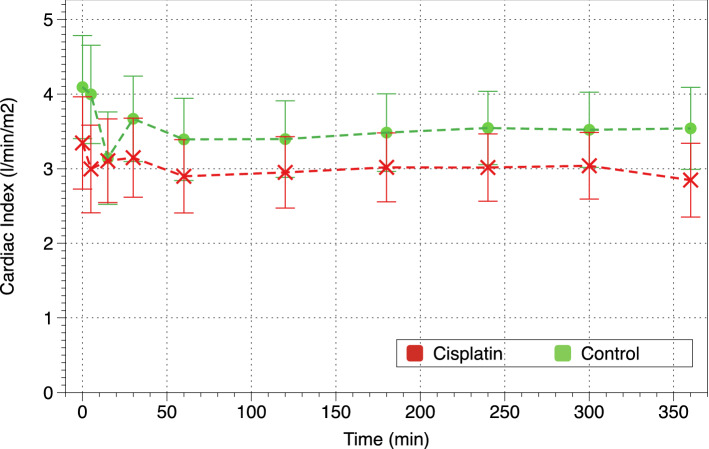
Evolution of cardiac index (CI).

**Figure 4:**
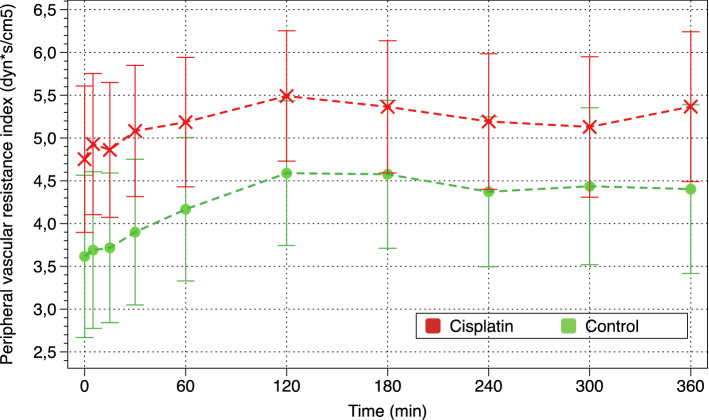
Evolution of systemic vascular resistance index (SVRI).

Cardiac index (CI) and systolic volume index (SVI) decreased in both groups, but they were lower in cisplatin piglets (p = 0.056 and p = 0.025, respectively) (Fig. 3). Cisplatin piglets had significantly higher peripheral/systemic vascular resistance index (PVRI) than control piglets (p = 0.007) at baseline and throughout the whole study. PVRI did not change significantly during the 6 h of CRRT, but they were significantly higher in cisplatin piglets than in the control group (Fig. 4).

There were no significant differences in GEDI, dPmax, SVV and CVP between groups (Table 2).

**Table 2:** Evolution of heart rate, central venous pressure, stroke volume index, global end diastolic index and temperature.

	Baseline	5′	15′	30′	60′	120′	180′	240′	300′	360′
	Mean (SD)	Mean (SD)	Mean (SD)	Mean (SD)	Mean (SD)	Mean (SD)	Mean (SD)	Mean (SD)	Mean (SD)	Mean (SD)
HR bpm	112 (22.4)	109 (30)	105 (23.4)	106 (23.4)	100 (19.5)	106 (22)	105 (22.6)	112 (23.2)	112 (30)	117 (23.5)
CVP cmH_2_O	9.6 (4)	9.7 (3.6)	10.2 (3.5)	9.9 (3.8)	9.1 (3.6)	8.9 (4.2)	9.1 (3.7)	9.1 (3.7)	9.2 (3.9)	9.3 (4)
SVI ml/m2	30.4 (9)	33.5 (9.7)	32.4 (7.6)	31.7 (7.7)	30.8 (8)	29.6 (8.3)	30.5 (7.7)	29.1 (7.6)	29.1 (7.7)	27 (7.7)
GEDI ml/m2	396.7 (155.2)	384.4 (160.3	386.2 (138.4)	369 (146.7)	366.3 (157.3)	328.2 (106)	337.2 (117.2)	331.3 (123.3)	327.6 (123.7)	304.2 (121.6)
Temp °C	38 (1.9)	37.5 (1.8)	37.4 (1.8)	37.2 (1.6)	37.1 (1.5)	37.1 (1.3)	37.5 (1.4)	37.9 (1.3)	37.9 (1.2)	38.1 (1)

*CVP *Central venous pressure, *GEDI *global end diastolic volume index, *HR *heart rate, *SVI *stroke volume index, *Temp *Temperature.

CVP: Differences in central venous pressure between baseline and 5 min: p = 0.760; baseline and 15 min: p = 0.218; baseline and 30 min: p = 0.542; baseline and 60 min: 0.359; baseline and 120 min p = 0.207; baseline and 180 min p = 0.690; baseline and 240 min p = 0.881; baseline and 300 min p = 0.604; baseline and 360 min p = 0.414.

GEDI: Differences in global end diastolic index volume between baseline and 5 min: p = 0.329; baseline and 15 min: p = 0.425; baseline and 30 min: p = 0.358; baseline and 60 min: 0.165; baseline and 120 min p = 0.019; baseline and 180 min p = 0.072; baseline and 240 min p = 0.028; baseline and 300 min p = 0.032; baseline and 360 min p = 0.019.

HR: Differences in heart rate between baseline and 5 min: p = 0.256; baseline and 15 min: p = 0.008; baseline and 30 min: p = 0.062; baseline and 60 min: 0.004; baseline and 120 min p = 0.271; baseline and 180 min p = 0.271; baseline and 240 min p = 0,197; baseline and 300 min p = 0.782; baseline and 360 min p = 9.580.

SVI: Differences in stroke volume index between baseline and 5 min: p = 0.096; baseline and 15 min: p = 0.142; baseline and 30 min: p = 0.306; baseline and 60 min: 0.829; baseline and 120 min p = 0.542; baseline and 180 min p = 0.883; baseline and 240 min p = 0.347; baseline and 300 min p = 0.388; baseline and 360 min p = 0.281.

Temp: Differences in temperature between baseline and 5 min: p < 0.001; baseline and 15 min: p < 0.001; baseline and 30 min: p < 0.001; baseline and 60 min: p < 0.001; baseline and 120 min p = 0.005; baseline and 180 min p = 0.150; baseline and 240 min p = 0.328; baseline and 300 min p = 0.943; baseline and 360 min p = 0.990.

### Biomarkers of acute kidney injury (AKI)

Table 3 shows baseline serum levels of renal function parameters (creatinine, urea and Cystatin C), urine kidney injury biomarkers (uNGAL, uKIM-1), serum ADH and urine aquaporin-2. All renal function parameters, as well as uNGAL and uKIM-1, were elevated in the cisplatin group of piglets. Creatinine, urea and cystatin C levels decreased significantly during CVVHDF in the cisplatin group (p < 0.001 and p = 0.003, respectively). This decrease was not observed in control piglets. uKIM-1 levels decreased significantly in both groups (p = 0.001), and there were no significant changes in uNGAL levels during CVVHDF.

**Table 3:** Baseline parameters and biomarkers of renal function and injury.

Baseline parameters	Control Mean (SD)	Cisplatin Mean (SD)	p
**Blood**			
Creatinine (mg/dl)	0.58 (0.08)	3.12 (0.98)	< 0.001
Urea (mg/dl)	38 (7)	166 (45)	< 0.001
Osmolality (mmol/l)	288 (4)	304 (7)	< 0.001
Sodium (mEq/l)	140 (2)	138 (3)	0.011
Cystatin C (ng/ml)	1.5 (0.8)	3.5 (2.8)	0.06
ADH (pg/ml)	140 (79)	82 (66)	0.22
**Urine**			
NGAL (ng/ml)	136.3 (40)	370 (153)	< 0.001
NGAL/uCr ratio	1.3 (1.1)	5.2 (4.4)	0.004
KIM-1 (ng/ml)	0.09 (0.04)	0.6 (0.55)	0.011
KIM-1/uCr ratio	0.002 (0.003)	0.009 (0.01)	0.085
Aquoporin-2 (ng/ml)	3.3 (2.2)	0.9 (0.35)	< 0.001
Aquoporin-2/uCr ratio	0.05 (0.06)	0.01 (0.001)	0.046

**Figure 5:**
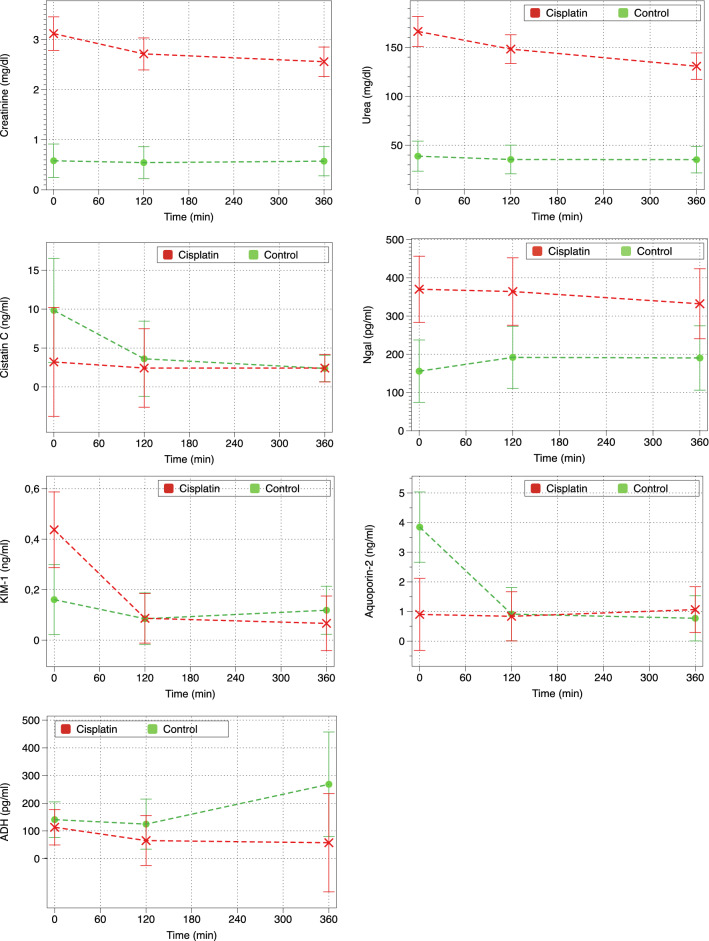
Evolution of renal function parameters and kidney biomarkers: serum creatinine, urea cystatin C and ADH and urine NGAL, KIM-1 and Aquaporin 2.

Aquaporin-2 levels were lower in cisplatin piglets at baseline (p < 0.001) and did not change throughout the experiment. Control piglets, however, had higher aquaporin-2 levels at baseline and suffered a significant decrease during CVVHDF (Fig. 5).

There were no significant differences in urine osmolality between cisplatin and control piglets [690 (115) vs 534 (113) mOsm/L, p = 0.787]. There were no significant differences in sodium excretion fraction (EFNa) [0.39 (0.25) vs 0.84 (0.8), p = 0.062] or in transtubular potassium gradient (TTKG) [5 (1.8) vs 11.7 (4.2), p = 0.213]. Solute load was greater in the cisplatin group of piglets [pOsm 304 (7) vs 288 (4) mOsm/L, p < 0.001], mainly due to elevated urea levels [166 (45) vs 39 (7) mg/dL, p < 0.001], as glucose, sodium and other solutes were similar in both groups of piglets.

## Discussion

We hypothesized that the cause of the decrease in urine output after starting CVVHDF was a reduction in RBF due to hemodynamic deterioration. However, the results of our study suggest that RBF is not responsible for the decrease in UO, as it significantly increased in both groups despite the unfavorable hemodynamic changes.

Transit time flowmeters have some advantages over electromagnetic flow probes or doppler flow probes in the clinical practice. Transit time flow measurement is fast and easy even under surgical conditions, artifacts affect results less severely and it is not affected by vessel size^16–20^. Measuring blood flow on the renal artery renders real-time information about global kidney perfusion, but it doesn’t differentiate between cortical and medullary perfusion. Even so, this study provides some valuable and novel information, as it reveals that CRRT causes changes in the renal vascular regulation system leading to an increase in renal blood flow in hemodynamically stable piglets. This finding opens the door to future research on the changes that take place during CRRT at the glomerular level.

RBF remained significantly lower in piglets with AKI. Winston et al.^21^ and Daugaard et al.^22^ also described significantly lower RBF in rats and dogs treated with cisplatin than in control animals. They conclude that the reduced glomerular filtration rate (GFR) in early (48–72 h) cisplatin-induced renal failure is accompanied by reversible changes in RBF and renal vascular resistance. They conclude that, in the early stages of cisplatin-induced renal failure (48–72 h), the reduction in glomerular filtration rate (GFR) is accompanied by reversible changes in RBF and renal vascular resistance.

Bedside Doppler ultrasound is increasingly being used as a surrogate marker of kidney perfusion in critically ill patients^23^. Our study showed a weak correlation between invasive (flowmeter on renal artery) and non-invasive indirect markers of kidney perfusion as RRI and PI at baseline. Nevertheless, RRI and PI were not able to detect the changes which took place in RBF during the study period. Similarly, Avasci et al. also described the limited ability of renal doppler parameters to detect changes of up to 50% in RBF^24^. Since invasive monitoring of RBF is not an option in the clinical setting, clinical decisions must be made upon such indirect measurements. Therefore, RRI and PI might be useful, but they shouldn’t be used as surrogate markers of RBF.

As reported in our previous study, a significant decrease in blood pressure, CI and PVRI occurred when starting CRRT in both groups^15^. No statistically significant differences were found in blood pressure between groups. Nevertheless, cisplatin piglets had a lower CI [even though it did not reach statistical significance (p = 0.056)], lower SVI and lower RBF than control piglets throughout the study. Therefore, even though both groups were hemodynamically stable at baseline, “sicker” piglets are, apparently, more susceptible to hemodynamic changes during CRRT than “healthy” piglets. This appreciation is also observed in the clinical setting, where unstable patients usually tolerate the connection worse than stable patients^10^.

Cisplatin piglets had higher PVRI at baseline than controls. Mean core temperature in cisplatin piglets at baseline was 37.1 °C (1.5 °C lower than control piglets). Housing conditions were similar for all the animals included in the study. During the experiment, active heating measures were applied equally to all animals in order to maintain normothermia. Active heating measures included an electric heating blanket under the piglet and the Prismacomfort blood heater in the return line. Normal body temperature for piglets is between 38.0 and 40.0 °C, so 37.1 °C is considered mild hypothermia. Mild hypothermia increases peripheral vascular resistances^25,26^. This could explain, at least in part, the differences in PVRI between groups. The FDA reports hypothermia induced by cisplatin as a rare complication (0.08%), which occurs early in the treatment and mainly in elderly patients. A Polish article published in 1995 also described hypothermia in 5 out of 11 patients receiving cisplatin for the treatment of lymphoma^27,28^. This complication was also described in an experimental study in rats, in which hypothermia was reported to occur in all rats treated with cisplatin^29^. In the light of our findings, piglets seem to have the same behavior as rats in temperature regulation when receiving cisplatin. Nevertheless, hypothermia cannot fully explain the differences in PVRI, because both groups had the same mean body temperature at the end of the study, but cisplatin piglets still had significantly higher PVRI than control piglets.

Cisplatin-based chemotherapy causes direct damage to the vascular endothelium^30^. In vitro exposure of endothelial cells to cisplatin or bleomycin causes cytokine release and cytotoxicity^31,32^. Markers of inflammation and endothelial dysfunction are also evident after cisplatin-based chemotherapy, including Von Willebrand factor, fibrinogen, tissue-type plasminogen activator, and high-sensitivity C-reactive protein^33,34^. This damage to the endothelium impairs the normal activity of endothelial nitric oxide synthase (eNOS), reducing the production of Nitric oxide (NO). NO is an important signaling chemokine in vascular homeostasis. It is a potent vasodilator, so a lack of NO will result in vasoconstriction and increased PVR. Cisplatin has been accused of inducing endothelial dysfunction by attenuating NO production in human umbilical vein endothelial cells ^35^.

Even though cisplatin piglets had higher PVRI than control piglets, there was a trend in PVR to decrease immediately after connection in both groups. The cause of this decrease in PVR can be due to an excess of vasodilatory agents or to a lack of vasoconstrictor agents (ADH, angiotensin). An excess of vasodilatory agents (NO, bradykinin, prostaglandins), perhaps in response to the contact of the blood with the extracorporeal CRRT circuit, has been proposed by several authors^36,37^. Unfortunately, we were not able to measure the levels of NO, bradykinin, prostaglandins, or the activity of the renin–angiotensin–aldosterone axis (RAA) in this study.

Arginine vasopressin (AVP), also known as antidiuretic hormone (ADH), also plays an important role in the regulation of vascular muscle tone. AVP binds to V1 receptors on the vascular smooth muscle increasing vascular tone. Inadequately low levels of ADH plasma concentrations are suspected to contribute to cardiovascular failure in vasodilator shock^38,39^. There are three interesting findings in terms of ADH in our study. In the first place, cisplatin piglets had lower levels of ADH. ADH plays an essential role in the physiology of water balance via the V2 receptor, resulting in aquaporin-2 (AQP-2) exposure at the apical plasma membranes of the collecting duct cells. AQP-2 is a water-selective channel expressed in mammalian kidneys and can regulate water reabsorption in the collecting duct cells. This regulation depends, mainly, on its expression and accumulation at the apical plasma membrane, and is regulated by the ADH-cAMP-AQP-2 signaling pathway^40,41^. It has been suggested that cisplatin decreases the expression of AQP-2 and decreases serum levels of ADH. This lack of water channels (AQP-2) and ADH would be responsible for the polyuria and urine concentration defect that occur in cisplatin-induced AKI^42,43^. As described in these studies, cisplatin piglets had lower ADH and AQP-2 levels. However, the piglets in our study were not polyuric, but had a lower UO than control piglets. We were unable to find an explanation for this finding.

Secondly, cisplatin piglets had lower levels of ADH but higher PVRI than controls. Therefore, ADH levels are not responsible for the increased PVRI found in cisplatin piglets.

In third place, baseline ADH levels were surprisingly high in all piglets (> 100 pg/ml). Normal ADH levels in humans range between 1 and 5 pg/ml. Cisplatin can alter ADH function causing both syndrome of inappropriate ADH secretion (SIADH) and diabetes insipidus ^44^. There were no other signs of SIADH as serum sodium and osmolality were normal in both groups of piglets. There are no references in the literature for normal ADH levels in pigs, but Gao et al. also described ADH levels of above 100 pg/ml in healthy rats ^45^.

Renal function parameters (creatinine, urea, cystatin C) were higher in cisplatin piglets, as expected, due to cisplatin-induced AKI.

Serum creatinine and urea are not direct markers of AKI, but surrogate markers of glomerular filtration rate (GFR). When GFR is reduced, p-creatinine will only increase after some time. This means that a timely diagnosis of AKI can be missed, especially when AKI is not associated with a significant reduction in GFR, as it usually occurs in early stages of nephrotoxic-induced AKI^46^. Novel kidney injury biomarkers such as NGAL and KIM-1 could offer some advantages over creatinine and urea in the immediate stage after the insult to the kidney, as they seem to be early indicators of kidney injury despite GFR^47–49^. Nevertheless, more studies are needed to support their usefulness in the clinical setting. On the other hand, at a steady state in AKI, changes in GFR will usually be reflected in p-creatinine. Samples in our study were taken 48 h after the renal insult (cisplatin administration). Both kidney injury (KIM-1 and NGAL) and GFR (creatinine and urea) markers were significantly higher in cisplatin piglets, but we are unable to tell which markers were more precocious. Urine NGAL/Creatinine and Aquaporin/Creatinine ratios were also significantly higher in the AKI group of piglets, so the differences between groups are not attributable to a lower urine output but to kidney damage in piglets with AKI.

There were no significant differences in urine osmolality, EFNa or TTKG between cisplatin and control piglets. Therefore, overall tubular function and the concentrating ability of the kidney was preserved despite kidney injury due to cisplatin.

Urine output would be expected to be higher in cisplatin piglets, as solute load was higher in this group of piglets. Nevertheless, urine output was much lower in this group of piglets. An increased ADH would explain the decreased urine output despite an increased solute load. Nevertheless, ADH levels did not differ from those of control piglets (they were, in fact, slightly lower than control piglets), and no differences were found in urine osmolality between groups. Therefore, the decreased urine volume can only be explained by the reduced glomerular filtration rate (in the absence of tubular disfunction). Perhaps the different behavior of ADH levels could determine a different mechanism for oliguria between groups: The significant increase in ADH levels in control piglets could account for a decrease in urine output. Nevertheless, uOsm values did not change during CRRT, and AQP-2 levels significantly decreased instead of increasing as expected by an increased ADH. On the other hand, ADH levels did not change in cisplatin piglets, nor did uOsm. Further studies are needed to fully understand the mechanisms of decreased urine output during CRRT.

Prowle et al. systematically reviewed the evidence for the association between RBF and GFR in AKI^50^. They included 22 studies and 250 patients. They conclude that there is only a limited association between estimated renal plasma flow and GFR, and no detectable association between total renal plasma flow (measured directly) and GFR.

Our study has several limitations. In the first place, the duration of CRRT in our study was only 6 h, so our results can only explain the changes occurring during this first phase of CRRT. Secondly, piglets were anesthetized during the study, which can affect hemodynamics and, perhaps, RBF. Nevertheless, piglets were stabilized during one hour before starting CRRT, so all the changes that were observed during the connection happened under the same sedation they had at baseline. Furthermore, critically ill patients undergoing CRRT are usually under sedation too, so the conditions in this study don’t differ that much from PICU clinical practice. In the third place, we were not able to measure bradykinin or other cytokines and hormones involved in vascular tone, therefore limiting our ability to understand the hemodynamic changes upon connection.

In the fourth place, animals were only weighed before the experiment. Another limitation is that some samples of novel AKI biomarkers (NGAL, KIM-1), ADH and AQP-2 were missing. The small sample size of these determinations reduces the statistical power and, therefore, the ability to detect significant differences in some analysis. Finally, we were not able to find normal reference values for some parameters (such as ADH and AQP2) in piglets.

## Conclusions

CVVHDF initiation produced a significant decrease in arterial blood pressure and cardiac index in both healthy piglets and in those with acute kidney injury. The exact mechanisms for these hemodynamic changes are still to be elucidated.

Renal blood flow increased after starting CVVHDF. Therefore, a reduced RBF is not the cause of the decrease in UO. Unfortunately, the cause of the decline in UO during CVVHDF remains an unanswered question.

More studies are needed to elucidate the exact mechanisms of the changes in hemodynamics and urine output during CRRT.

## Methods

We carried out a prospective randomized experimental study in 2–3 month old Maryland minipigs weighing between 9 and 11 kg. The experimental protocol was approved by the Gregorio Marañon University Hospital Ethics Committee for Animal Research (4-2/2012). Animal studies were conducted in the Experimental Medicine and Surgery Unit of the Gregorio Marañón University Hospital in Madrid, Spain. Animal care was carried out by qualified staff, and international guidelines for ethical conduct in the care and use of experimental animals were applied throughout the study.

Piglets were randomized into two groups: piglets with non-oliguric acute kidney injury and healthy (control) piglets. The Epidat 4.2 program for epidemiologic data management was used for randomization (Epidat: programa para análisis epidemiológico de datos. Versión 4.2, julio 2016. Consellería de Sanidade, Xunta de Galicia, España; Organización Panamericana de la Salud (OPS-OMS); Universidad CES, Colombia).

The model described by Santiago et al. was used to induce a non-oliguric AKI^15^. Piglets were pre-medicated with intramuscular ketamine (15 mg/kg) and atropine (0.02 mg/kg) before inserting a peripheral vein catheter in the ear to administer a dose of cisplatin (3 mg/kg) or placebo (control group).

Forty-eight hours later, animals were anesthetized with boluses of propofol (5 mg/kg), fentanyl (5 μg/kg) and atracurium (0.5 mg/kg) for oral endotracheal intubation. Sedation and muscle relaxation by continuous infusion of propofol 10 mg/kg/h, fentanyl 10 mcg/kg/h, and atracurium 2 mg/kg/h were maintained throughout the procedure.

Once piglets were intubated and sedated, arterial and venous central lines were inserted. Cannulation of femoral arterial (4F PiCCO catheter [PiCCO1, Pulsion Medical System, Munich, Germany]) and venous (three-lumen 5F catheter) accesses was ultrasound-guided.

Blood pressure was continuously monitored via the arterial catheter inserted in the femoral artery and displayed in the PiCCO monitor.

The PiCCO monitor uses 2 different methods for measuring cardiac output: pulse contour analysis of the arterial pressure waveform and transpulmonary thermodilution.

The transpulmonary thermodilution method requires the infusion of 5 ml of cold normal saline through the central venous catheter. The arterial line measures the dop in blood temperature. The monitor then applies the Stewart–Hamilton equation to the area under the thermodilution curve obtained from the changes in blood temperature to calculate cardiac output. Pulse contour analysis renders continuous information of cardiac output, as it provides continuous beat by beat parameters which are obtained from the shape of the arterial pressure wave. The area under the arterial curve during systole, minus the background diastolic area, is assumed to be proportional to the stroke volume. Cardiac output is then derived from the stroke volume and heart rate. Pulse contour analysis requires an initial calibration with transpulmonary thermodilution^51,52^.

An 8 F double lumen dialysis catheter was surgically inserted in the external jugular vein.

An ultrasonic transit-time flow probe (HDQ1.5FSB, Transonic Systems Inc., Ithaca, New York, USA) was placed on the left renal artery for continuous renal blood flow monitoring, as described in our previous study^15^. Bladder was directly catheterized through a suprapubic cystostomy to assess UO. Blood gases were analyzed using the GEM Premier 30001 blood gas analyzer (Instrumentation Laboratory, Lexington, Kentucky, USA).

Continuous veno-venous hemodiafiltration (CVVHDF) with a Prismaflex (Baxter Int.) monitor and a HF-20 filter (0.2 m^2^) was started after a 30-min stabilization period. CVVHDF was started according to the connection protocol used in our unit, with a slow initial blood flow of 20 ml/min and 10 ml/min increases every minute until a goal blood flow of 5 ml/kg/min. Dialysis and substitution flow rates were then fixed at 20 ml/kg/h and fluid removal was adjusted for a neutral balance. All fluid intake and output, including UO, was considered for calculating water balance. A bolus of 150 UI/kg of non-fractioned heparin (UFH) was administered before the filter at the beginning of the therapy followed by a continuous infusion of 30–50 UI/kg/h.

The following parameters were recorded in the data sheet at baseline and at 5, 15, 30, 60, 120, 180, 240 and 360 min of therapy: electrocardiogram (ECG), temperature, heart rate (HR), systolic arterial pressure (SAP), mean arterial pressure (MAP), diastolic arterial pressure (DAP), cardiac index (CI), central venous pressure (CVP), systemic vascular resistance index (SVRI), stroke volume index (SVI), systolic volume variation (SVV), global end-diastolic volume index (GEDI), maximum pressure difference (dPmax), transcutaneous oxygen saturation (HeartStart XL + 1, Philips Medical Systems, Andover, Massachusetts, USA), cerebral and renal regional oxygen saturation (INVOS™ 5100C Cerebral/somatic Oxymetry) and renal blood. Lowest parameters during the connection process were also recorded.

UO was measured hourly. Blood and urine tests were drawn at baseline and at 120 and 360 min of therapy.

Blood tests included osmolality, creatinine, urea, uric acid, glucose, sodium, chloride, potassium, calcium, magnesium, AST, ALT, amylase, lipase, cystatin C and ADH. Urine tests included osmolality, creatinine, urea, uric acid, glucose, sodium, chloride, potassium, calcium, magnesium, NGAL, KIM-1 and aquaporin 2.

Ultrasound of the right kidney was also performed at baseline, 30 min, 60 min and then hourly until the end of the study. Ultrasound measurements were standardized as follows:

A 2–5 MHz ultrasound probe was placed in the right renal fossa using the B mode gray-scale sonography. Once the right kidney was identified, color mode was activated to identify renal vessels. Target arteries for renal resistive index (RRI) sampling are the arcuate or interlobar arteries, adjacent to medullary pyramids. Once the target vessel is identified, the “Pulsed Wave Doppler” mode is activated, selecting a doppler window frame of 2–5 mm. The angle of the ultrasound beam must be corrected in order to align it with the vessel. In order to maximize waveform size, care should be taken in using the lowest pulse repetition frequency without aliasing, the highest possible gain without noise and the lowest wall filter. When at least 3 reproducible waveforms have been obtained, the image was frozen and the “Measurement” button was selected to calculate RRI and PI (pulsatility index) of each wave, and mean RRI and PI was calculated for the right kidney.

Piglets were euthanized at the end of the experiment by administering supra-anesthetic doses of fentanyl and propofol immediately followed by a rapid intravenous infusion of potassium chloride (4 mEq/kg).

Stata^®^ 14 data analysis and statistical software was used. The mixed-effects model was used to assess the behavior of the different variables over time and to compare the differences between groups**.** The Spearman correlation model was used to assess associations between different variables. A p value of < 0.05 was considered statistically significant.
